# Debility and Diarrhœa of Soldiers

**Published:** 1863-02

**Authors:** 


*Debility and Diarrhœa of Soldiers.*—Rufus K. Browne, M. D., Surgeon in charge of the U. S. Marine Hospital recommends (*Med. Times*) in debilitated but not particularly anæmic state of patient: ℞—Cinchon. Rubr. ℥ ij; Anthem. ℥ ss; Fl. Ext. Gentian. ʒ ij; Tr. Aurant. Cort. ℥ ij; Sodae Bicarb. ʒ ij; Aq. ʒ xxxij. M. Boil twelve minutes and strain. D. Half a teacupful every two hours. He sometimes substitutes Tinct. Colombo or Quassia for the Anthemis. If the patient be greatly enfeebled, as the result of protracted sickness or diarrhœa, he orders each dose of the above to be followed by an ounce of Santa Cruz or Jamaica Rum with five grains of Ginger. Or if anæmic withal, administers a grain of Pulv. Strychnos nux. vom. and five grs. Pot. Tart. Ferri. He believes the latter the most energetic replenisher of the active elements of the blood.

To abbreviate the chill in periodic diseases, he orders an ounce of Rum, with four gr. Capsic. and three of Ginger, in *hot* water. A mixture of Squills and Verat. Virid. has been found useful in pneumonia and other affections where the pulse is high. By it, he says, "you rapidly modify the circulation, but there its efficacy ends."

A solution of veratria in glycerine is convenient and efficacious in removing superficial pains.

As an "unfailing and undisturbing evacuant, he directs: ℞ Ol. Ricin. Com. ℥ss.; Ol. Olivœ ℥ss.; Ol. Tiglii gtt. j; Es. Ol. piper. menthæ gtt. iv M. Or, according to indications, Calomel may be preceded or added to it. He prefers this combination to the salines in treating military diarrhœa.

Where salines are used, he prefers the Tartrate of Soda; and where there are fæces in the large intestine he commends: ℞ Aloes Socot. gr. xx; Acid Sulph. dilut. gtt. x M. f. p. IV. One to be taken every hour until discharged. "However long the constipation, this will always relieve the bowels with very little or no sense of its *effect*."

I have sometimes arrested the continuance of this exhausting form of diarrhœa, when every other article in the hospital dispensary had failed, by

℞ Pulv. resinæ communis gr. viij. or x., given in water, pulv. acaciæ, or even powder of arrow-root may be added to this, if the discharges are streaked with blood.

In that case also the following:

℞. F. ex. gentian. ʒij. F. ex. senegæ ʒj. Syr. ipecac ʒij. Gum. acaciæ ʒi. Aquæ ℥ij. M. A teaspoonful every two hours. The senega is a restorative; *how*, I do not know. I have invariably found it a useful adjunct in tonic mixtures. The nearest a uniformly efficacious diarrhœa powder, and generally efficacious where the diarrhœa has not been long uninterrupted, is ℞. Pulv. gum. acaciæ ʒij. Cretæ prep. ʒj. Pulv. kino ʒi. Pulv. ipecac. gr. v. Div. in p. æq. No. xx., one after each discharge. This with mucilaginous drink, where there is torpidity of the bowels, in the highly enfeebled, if we suspect but little fæcal contents, and dread any disturbance of the system, and the stomach and perhaps bowels are weak.

℞. Ol. ricini ℥i.; Ol. morrhuæ ℥ss.; Gum. acaciæ ʒij.; Aquæ menth. pip. ʒiv.; Tr. opii gtt. xl.; Spts. terebinthinæ gtt. xxv. A tablespoonful every two hours. It is particularly useful where there had previously been intestinal capillary hæmorrhage, or where there is continued intestinal torpidity, and the patient is confined to his bed.

In moderately reduced states of the system, where there is almost a daily recurrence of slight febrile symptoms without rigor, give ℞. Tr. serpentariæ ℥j.; Tr. rhei ℥j.; Tr. zingiberis ℥j.; F. ext. valerian. ℥j. Tablespoonful every two hours. The nervine in combination with the ginger is a particularly kind but efficacious queller of the febrile temper. I have never found any particular benefit from serpentaria alone. Of that and in such a case the tincture is better than the infusion. For immediate and entire subsidence of flatulent colic or pain, ℞. Liquor, ammon., Chloroform—*aa.* gently rubbed over the epigastrium is particularly efficacious.

